# Joining forces for pediatric very rare tumors

**DOI:** 10.18632/oncotarget.26871

**Published:** 2019-05-03

**Authors:** Andrea Ferrari, Dominik T. Schneider, Gianni Bisogno, Annalisa Trama

**Affiliations:** Annalisa Trama: Department of Epidemiological Research and Molecular Medicine, Evaluative Epidemiology Unit, Fondazione IRCCS Istituto Nazionale dei Tumori di Milano, Milano, Italy

**Keywords:** adolescents, cancer, story, youth project, model

Dealing with rare tumors remains a huge challenge for oncologists worldwide, even in 2019. The reasons are multifaceted and concern every step of their management, from epidemiology and etiology to diagnosis and treatment. Pediatric cancers include a particular group of rare tumors, and it seems worthwhile to report on lessons we learned in recent years, and in particular how it becomes clear that all stakeholders—not just oncological physicians and researchers, but also epidemiologists, IT experts, patients and their families, regulatory authorities and international funding bodies—need to join forces to face this challenge.

All childhood cancers are rare and that is why pediatric oncologists have put a lot of effort into developing national and international cooperative clinical and research protocols, which have enabled us to pool our understanding of pediatric tumors, and improve patients’ chances of survival as a result. But there is a set of pediatric neoplasms, the so-called very rare tumors (VRTs), that are so rarely encountered that patients have not benefited from the historical achievements of large-scale pediatric oncology networking. These pediatric VRTs comprise a very heterogeneous assortment of diseases, some specific to childhood (such as pleuropulmonary blastoma or pancreatoblastoma), others common in adulthood but extremely unusual in pediatric age (like colon cancer and melanoma), with biological and clinical features that distinguish them from their adult counterpart [1].

In the past, pediatric VRTs aroused little interest because their rarity made them nearly impossible for researchers to generate results of any value within a reasonable time span. The young patients involved sadly paid the price for the rarity of their tumors and the shortage of dedicated studies, or even treatment guidelines (patients were always treated on an individual basis).

This situation changed in the first decade of the new millennium: pediatric VRTs began to attract the attention of major pediatric cancer reference centers, and cooperative groups of researchers. The first step forward saw the development of national cooperative programs, starting with the pioneering experience of the Italian TREP (*Tumori Rari in Età Pediatrica*, Rare Tumors in Pediatric Age) project [2], and culminating with the birth of the VRT network called EXPeRT (or the European Cooperative Study Group for Pediatric Rare Tumors). The EXPeRT was established in 2008 by the national VRT working groups from Italy, France, Germany, Poland, and the United Kingdom, its stated aim being to improve the available treatments and promote research in the relatively uncharted territory of pediatric VRTs [3].

The EXPeRT’s founding members had several things on their wish list: (1) to pool national retrospective series of specific tumor types to obtain large series enough to enable treatment-related risk stratification and generate homogeneous therapeutic recommendations, a shared research methodology, and a common framework [4–9]; (2) to develop an organization with the double purpose of promoting research and serving as an advisory network to help with difficult decisions regarding single clinical cases; and (3) to set up an international prospective case registry.

With support from the European pediatric oncology community (i.e. the European Society for Pediatric Oncology, SIOPE), the EXPeRT network demonstrated that synergic, multi-level cooperation is the way to develop good-quality programs tailored to children who have, until recently, been rather neglected. It soon became clear that it was crucially important to obtain dedicated funds, and also to engage with figures outside the world of pediatric oncology, and the EXPeRT network played a large part in obtaining EU-funded projects.

It is worth mentioning three important collaborative efforts that have been undertaken.

First of all, the EXPeRT was part of the 3-year ExPO-r-Net (European Expert Paediatric Oncology Reference Network for Diagnostics and Treatment) project that ran from 2014 to 2017. This project was developed under an EU directive aiming to reduce inequalities across EU Member States: it focused on the need to improve access to highly specialized health care, particularly for patients with low-prevalence diseases requiring special expertise. As part of the ExPO-r-Net project (and thanks to the financial support it provided), the EXPeRT went to work to develop harmonized treatment guidelines for pediatric VRT, and to implement two e-Health schemes to facilitate the exchange of information and knowledge *via* internet rather than move patients. One scheme revolves around a website (www.raretumors-children.eu) for families and the non-scientific community. The other is a virtual online tumor board and advisory desk dedicated to professionals who need expert medical advice (http://vrt.cineca.it/). The ExPO-r-Net project and the EXPeRT’s activities became a model for a subsequent, broader project to create the ERN PaedCan (European Reference Network for Paediatric Cancer) (http://paedcan.ern-net.eu/).

Second (from 2016 to 2019), the EXPeRT was involved in the JARC (Joint Action on Rare Cancers) (http://www.jointactionrarecancers.eu/), promoted by the European Commission. Working with the JARC provided the perfect opportunity to: (1) put in place a cooperation between pediatric oncologists and oncologists dealing with rare tumors in adults; and (2) establish links with the world of epidemiology (and big numbers) essential to research on extremely rare tumors. In cooperation with the JARC, the EXPeRT promoted an effort to arrive at a consensus on the definition of pediatric VRTs (i.e., pediatric tumors with an annual incidence < 2/1,000,000 population) and to draw up a list of these conditions, which can serve as a starting point for prioritizing research on such tumors, based on patients’ real clinical needs [10].

Third, as part of the ERN PaedCan project, the EXPeRT is involved in the 2018-2021 PARTNER (Paediatric Rare Tumours Network, European Registry) scheme, the main purpose of which is to set up a European registry of pediatric VRTs, a common platform for registering cases. This will involve: harmonizing data from existing national registries, with the aid of novel IT tools; providing a registry for those European countries that do not already have one (thereby including in the process countries with a low average expenditure on health); linking the EU registry to the EXPeRT virtual consultation system.

The promotion of research on pediatric VRT (and thereby improving the prognosis of affected patients) is a challenging endeavor. It demands the input of new ideas and the activation of new partnerships every step of the way. The only solution is to join forces, pool our resources, boost our collaborative efforts, involve everyone working on pediatric VRTs, be they international experts, specialists in various fields, care providers, families and patients, regulators, or providers of funding. We also need to work in synergy with the adult oncology community, for which our pediatric collaborative experience may serve as a helpful organizational model.

We have learned a lot from working on pediatric VRTs, and plan in future to extend this collaborative process to more and more centers, to ensure that more and more patients can benefit from our pooled expertise. The clinical aspects of pediatric VRTs will be linked more and more effectively with clinical databases and population registries (given the potential impact of future research hinging on big data in this sector), and with biological data (exploiting virtual biobanks, for instance).
